# Refining our policies on co-authorship requirements

**DOI:** 10.1038/sdata.2017.133

**Published:** 2017-10-17

**Keywords:** Publishing, Research data

Since launch, *Scientific Data* has had a policy of not considering submissions describing datasets with co-authorship requirements—requirements that downstream users include the data generators as authors on any subsequent publications. Such requirements were perceived as conflicting with *Scientific Data*'s strong open science stance, and with our view that the proper way to attribute data generators is through citation of their datasets and their related publications. We are also concerned that these requirements can discourage data reusers from publishing re-analyses that conflict with interpretations or interests of the data generators. For sensitive human datasets, we have allowed controls needed to protect patient privacy, but not restrictions designed to prevent competitive re-analysis or ensure special authorship rights for the data generators.

In 2016, however, we were approached by a group of authors seeking to publish a large metabolomic dataset derived from the cohort managed by the Alzheimer’s Disease Neuroimaging Initiative (ADNI, http://adni.loni.usc.edu/). ADNI data users are required to list the initiative on the author byline, with the phrase ‘for the Alzheimer’s Disease Neuroimaging Initiative’ and must include a statement recognizing that the ADNI investigators contributed only to the design and implementation of ADNI, but did not participate in analysis or writing of this report. Arthur Toga, writing on behalf of ADNI, has described this as ‘an acknowledgment on the author line’, and not a co-authorship requirement *per se* (Arthur Toga is also a member of *Scientific Data*’s Editorial Board). ADNI’s success in promoting reuse of these datasets is undeniable. ADNI’s data have been used in more than 700 publications (http://adni.loni.usc.edu/news-publications/publications/), and by many independent groups who are not part of the ADNI investigator group (see also ref. 1).

We sought feedback from several independent members of our Editorial Board regarding this reuse condition, and co-authorship requirements more broadly. Board members from the human genomics community tended to express the clearest opposition to co-authorship requirements, where the landmark Bermuda and Fort Lauderdale agreements have established an expectation that large-scale genomic resource projects release their data in an open and unrestricted manner, without special authorship requirements (see ref. 2 for an overview). Nonetheless, the majority of the members we consulted ultimately recommended that we maintain some flexibility on this issue. Those members who looked closely at this specific case tended to encourage us to consider this submission, based on the wording of this author-line acknowledgment requirement and ADNI's proven history of encouraging data sharing and reuse in a difficult area. We ultimately followed this advice, and the final Data Descriptor was published today^3^.

Going forward, we will be giving our Editorial Board more flexibility to consider submissions with special acknowledgement requirements on a case-by-case basis, when:

- The data are derived from human cohorts, where a controlled-access system is required to protect participant privacy and where on-going management of the cohort and data use compliance verification merits the added level of acknowledgement.
- The authors can convincingly demonstrate that the requirements will allow competitive and critical re-use of the data. We will continue to decline datasets with co-authorship or collaboration requirements that are perceived by our board as conflicting with competitive reuse.

Regardless of whether special acknowledgement requirements exist, our authors will still be asked to formally cite the datasets described or used in their publications, and we will continue to encourage authors to follow this same practice at other journals that support data citation.

We hope that this compromise will allow us to continue to work for more transparent sharing of sensitive human datasets, a difficult area where flexibility is required, while still protecting our strong commitment to the principles of open science. We will continue to periodically review our policies on this issue, especially as formal data citation is more widely adopted in the scientific community.

## References

[b1] Weiner M. W. *et al.* Impact of the Alzheimer's Disease Neuroimaging Initiative, 2004 to 2014. Alzheimer's & Dementia 11, 865–884 (2015). 10.1016/j.jalz.2015.04.005 PMC4659407 26194320

[b2] Kaye J., Heeney C., Hawkins N., de Vries J. & Boddington P. Data sharing in genomics—re-shaping scientific practice. Nature Reviews Genetics 10, 331–335 (2009). 10.1038/nrg2573 PMC2672783 19308065

[b3] St John-Williams L. *et al.* Targeted metabolomics and medication classification data from participants in the ADNI1 cohort. Sci. Data 4, 170140 doi: 10.1038/sdata.2017.140 (2017). 10.1038/sdata.2017.140 PMC5644370 29039849

